# Plasmapheresis facilitates soluble BCMA clearance and contributes to reversing primary resistance to anti-BCMA immunotherapy in multiple myeloma

**DOI:** 10.1038/s41375-025-02757-6

**Published:** 2025-09-10

**Authors:** Sven Neubert, Umair Munawar, Julia Mersi, Julia Noderer, Silvia Nerreter, Shilpa Kurian, Seungbin Han, Christina Verbruggen, Emma Besant, Nina Rein, Max Köppel, Johanna Lehmann, Tabea Köhler, Hannah Labinsky, Sigrun Häusl, Yoko Tamamushi, Xiang Zhou, Jule Pinter, Anna Laura Herzog, Kai Lopau, Elion Hoxha, Christoph Rummelt, Elena Gerhard-Hartmann, Andreas Rosenwald, Torsten Steinbrunn, Thomas Nerreter, Michael Hudecek, Hermann Einsele, Leo Rasche, K. Martin Kortüm, Johannes M. Waldschmidt

**Affiliations:** 1 Department of Internal Medicine II, University Hospital of Würzburg, Würzburg, Germany; 2 Department of Internal Medicine I, University Hospital of Würzburg, Würzburg, Germany; 3 Department of Internal Medicine I, University Hospital of Freiburg, Freiburg, Germany; 4 Institute of Pathology, University of Würzburg, Würzburg, Germany; 5 Department of Medical Oncology, Dana-Farber Cancer Institute, Harvard Medical School, Boston, MA USA; 6 Mildred-Scheel-Nachwuchszentrum (MSNZ), University Hospital of Würzburg, Würzburg, Germany; 7 Interact Advanced Clinician Scientist Program, University Hospital of Würzburg, Würzburg, Germany

**Keywords:** Cancer immunotherapy, Myeloma

Bispecific antibodies against the B-cell maturation antigen (BCMA) exhibit potent single-agent activity in multiple myeloma (MM), yet not all patients respond with a cumulated primary resistance rate of up to 40% of patients across all trials and agents [1]. While resistance has primarily been associated with antigen loss by acquired deletions or mutations [2–4], epigenetic target inactivation [5, 6], T-cell exhaustion [7] and an immunosuppressive microenvironment at baseline [8], translational data from the MajesTEC-1 trial indicates that elevated plasma levels of soluble BCMA (sBCMA) in MM patients with high tumour burden may impair antibody binding and can result in a dysfunctional effector:target (E:T) ratio [9]. This study by Lee and colleagues further dissected that a stepwise increase of sBCMA levels blocks the specific binding capacity of BCMA× CD3 bispecific antibodies in vitro, ultimately leading to complete resistance. Clinical validation data from MajesTEC-1 confirmed these findings in *n* = 147 relapsed/ refractory MM (RRMM) patients and established a significant relationship between baseline sBCMA levels and response to the BCMA×CD3 bispecific antibody teclistamab, with a median baseline sBCMA of 54.9 ng/mL and 304 ng/mL in responders and non-responders, respectively [10]. This led to the definition of a proposed sBCMA threshold of 400 ng/mL to infer baseline sensitivity to teclistamab [9]. Interestingly, the authors could also demonstrate that this effect can be partly overcome by increased E:T ratios, as the in vitro killing capacity of bispecific antibodies increases proportionally with a relative increase of effector T cells irrespective of sBCMA levels.

In this pilot study, we aimed to translate these findings to the clinic by exploring multimodal strategies to reduce plasma sBCMA levels in a 67-year-old patient with IgA kappa MM who was repetitively exposed to teclistamab over a period of 9.0 months. Informed consent was provided in line with the Declaration of Helsinki and local ethical standards (approval #08/21, *University Hospital Würzburg*). Serial sBCMA measurements were conducted from the peripheral blood (PB), bone marrow (BM) and ultrafiltrate (UF) removed during plasmapheresis (Fig. 1A), using a commercially available human ELISA-based quantification kit (ab263875, Abcam).

**Fig. 1:**
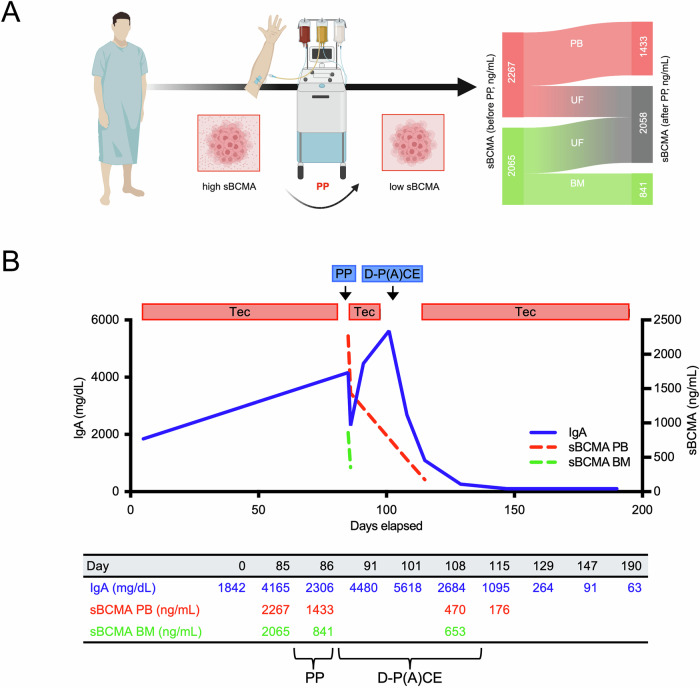
Multimodal debulking results in sBCMA clearance and subsequent response to BCMA× CD3 bispecific antibodies. **A** Design of study including sBCMA-levels (ng/mL) in peripheral blood (PB), bone marrow (BM) and ultrafiltrate (UF) before and after plasmapheresis. **B** IgA levels in PB (mg/dL) and sBCMA levels (ng/mL) in BM and PB over the course of treatment, graphical view (upper panel), tabular view (lower panel). Abbreviations: Tec, teclistamab, PP plasmapheresis, D-P(A)CE daratumumab, cisplatin, cyclophosphamide, etoposide. Figure 1A *created in BioRender under license* https://BioRender.com/9gtlmss.

Despite standard risk cytogenetics (del14p, gain16q, t(11;14)) at initial diagnosis, our patient exhibited functional high-risk disease illustrated by an early relapse ten months after D-VTd induction and autologous stem cell transplantation. Teclistamab was administered as second-line treatment, but no response or signs of cytokine release syndrome were observed. Suspecting primary resistance, we performed sBCMA quantification and detected substantially elevated levels of 2267 ng/mL and 2065 ng/mL in the PB and BM, respectively, along with a rising IgA of 4073 mg/dL (Fig. 1B). To address hyperviscosity symptoms, one session of plasmapheresis was performed. Tolerability of plasmapheresis was poor due to increased intravascular oncotic leakage, but led to the removal of a total of 8.5 mg of sBCMA within the UF, and a 36.8% and 59.3% reduction in PB-sBCMA and BM-sBCMA, respectively. Despite this reduction, sBCMA levels in both, PB (1433 ng/mL) and BM (841 ng/mL), remained above the MajesTEC1-defined threshold for teclistamab responders. In line with this data, our patient did not respond to second teclistamab exposure immediately after plasmapheresis. One cycle of debulking chemotherapy with D-P(A)CE (daratumumab- cisplatin- cyclophosphamide- etoposide without doxorubicin) was added. Soluble BCMA in PB subsequently dropped to 470 ng/mL and fell below the 400 ng/mL threshold (176 ng/mL) 14 days after the start of debulking chemotherapy. The patient was re-exposed to single-agent teclistamab 28 days after D-P(A)CE, showing a sixfold increase in IL-6 levels, but no clinical signs of cytokine release syndrome. Remission deepened over time, with the M-spike decreasing from 38.7 g/L to undetectable, consistent with a very good partial response as per IMWG criteria. Teclistamab was terminated after 6.3 months due to a paraosseus extramedullary relapse. The patient was re-exposed to salvage chemotherapy, but showed no response. At the last follow-up, three months after discontinuing teclistamab, he undergoes screening for an anti-GPRC5D (G-protein coupled receptor class C group 5 member D) based clinical trial.

We next aimed to recapitulate the impact of increasing sBCMA plasma concentrations on anti-BCMA immunotherapies in a firefly luciferase-expressing OPM-2 in vitro cell line model. OPM-2 cells were co-cultured with CD4⁺ CAR T-cells (cilta-cel (ciltacabtagene autoleucel), in-house BCMA-directed CAR-Ts) at an E:T ratio of 4:1. For teclistamab, co-cultures were established using 10 nM teclistamab and pan-T cells from a healthy donor at a 4:1 E:T ratio. Additionally, we tested the anti-BCMA drug conjugate belantamab-mafodotin (belamaf) at a dose of 500 ng/mL. For all agents, sBCMA was titrated into co-cultures at concentrations ranging from 0 to 1000 nM. We could confirm data by Lee and colleagues [9] by showing loss of cytolytic efficacy for anti-BCMA-directed cellular therapies (cilta-cel, anti-BCMA CAR-T) with increasing sBCMA concentrations (Supplementary Fig. 1). Similarly, T cell activation, measured by interleukin-2 (IL-2) and interferon-gamma (IFN-γ) secretion in respective supernatants, declined for cilta-cel and anti-BCMA CAR-Ts with increasing concentrations of sBCMA (both *P* < 0.0001, Fig. 2A, B). For teclistamab, rising sBCMA levels significantly reduced IL-2 production (*P* < 0.0001), whereas IFN-γ was less affected (*P* = 0.006, Fig. 2A, B). In belamaf models IL-2 and IFN-γ secretion were overall measured at very low level and not impacted by increasing sBCMA concentrations as expected (*P* = 0.99, Fig. 2A, B). Yet, belamaf efficacy equally deteriorated with increasing sBCMA concentrations, ultimately resulting in a complete loss of its cytotoxic activity (*P* < 0.0001, Fig. 2C).

**Fig. 2:**
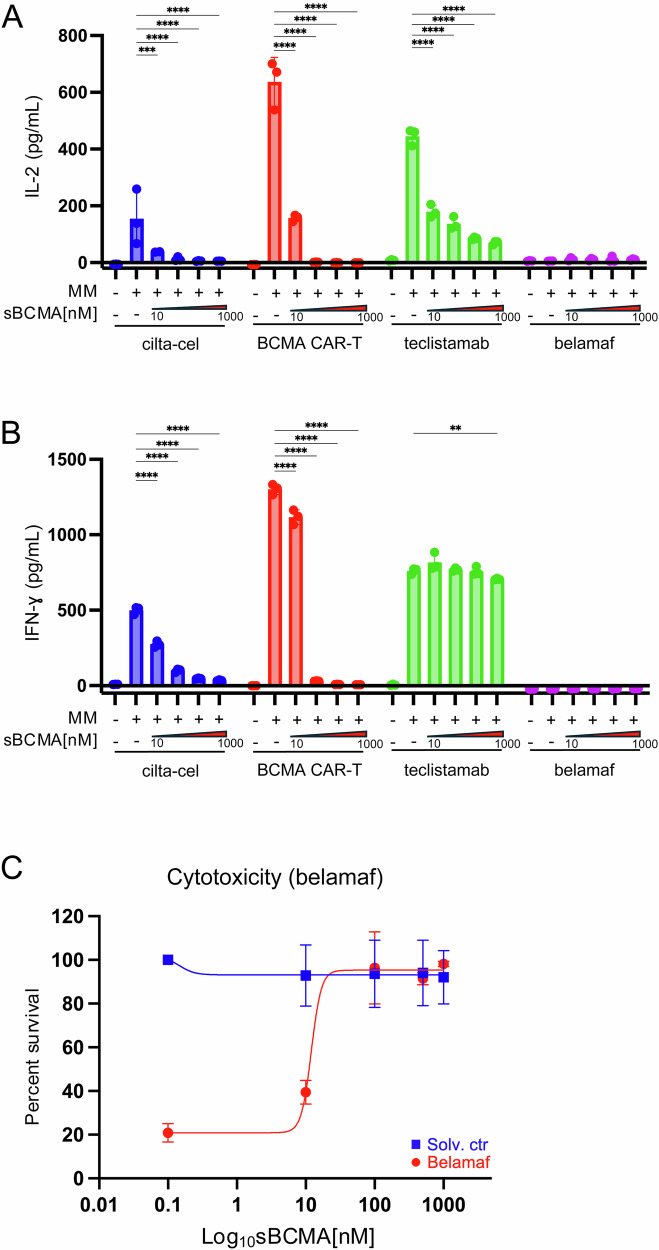
sBCMA limits in vitro efficacy of anti-BCMA immunotherapies. **A** IL-2 (pg/mL) and **B** IFN-γ (pg/mL) levels were quantified by ELISA from co-culture supernatants after 20 h of incubation using ELISA MAX Deluxe Set for human IL-2 (431816, BioLegend) and human IFN-γ (430116, BioLegend). **C** Cytotoxic activity of belamaf was assessed in the presence of sBCMA using a luminescence-based viability assay with PBS as solvent control (**P < 0.005, ***P < 0.001, ****P < 0.0001). Abbreviations: IL-2 interleukin-2, IFN-γ interferon-gamma, cilta-cel ciltacabtagene autoleucel, BCMA B-cell maturation antigen, CAR chimeric antigen receptor, belamaf belantamab-mafodotin, solv. Ctr solvent control.

In summary, this data confirms the central role of sBCMA in inducing primary resistance to anti-BCMA immunotherapies in MM. To the best of our knowledge, this is the first report on the feasibility of plasmapheresis as an additional debulking measure to lower sBCMA levels in PB and BM in the scenario of high tumour burden. Although chemotherapy may have interfered with the isolated impact of plasmapheresis on sBCMA reduction in our patient, this data underlines the need to integrate patient-tailored sBCMA monitoring and timely debulking strategies into clinical practice to facilitate meaningful responses in a larger fraction of MM patients treated with anti-BCMA immunotherapies. Our observations align with findings from the phase-2 MagnetisMM-3 trial, in which overall response to the BCMA×CD3 bispecific antibody elranatamab was only moderately reduced in ISS I–II patients with high-risk cytogenetics, penta-drug refractory disease or extramedullary disease (71.4%, 63.6% and 47.4%, respectively), while all patients with high tumour burden (ISS stage III) were primarily refractory [11]. Building on the previous concept established by Lee and colleagues, our data indicates that high sBCMA may also impair sensitivity to T-cell agnostic BCMA immunotherapies such as belamaf. Belamaf has previously been tested in combination with the γ-secretase inhibitor nirogacestat as part of the DREAMM-5 trial platform with the rationale to overcome BCMA antigen shedding and to increase longer treatment responses [12]. Ongoing clinical trials (NCT05090566, NCT04722146) of BCMA×CD3 bispecific antibodies combined with nirogacestat show promising early efficacy, though safety remains concerning with reported treatment-emergent mortalities of up to 21.4% in the MajesTEC-2 trial cohort C, including treatment-related cases of septic shock, COVID-19 and cardiac arrest [13]. To move forward, dual targeting approaches against BCMA and GPRC5D on the surface of MM cells, may depict the most promising strategy to overcome sBCMA abundance and antigen escape, as demonstrated by an interim update of a phase-1 study (NCT05652335), testing JNJ-5322 as the first-in-class BCMA×GPRC5D×CD3 trispecific antibody in triple-class exposed MM. This study demonstrated a highly remarkable ORR of 100% (89% ≥VGPR) at the recommended phase II dose in anti-BCMA/-GPRC5D naïve patients [14], and this concept is now being taken forward by innovative phase-2 trial concepts, including the MajesTEC-5/GMMG-10/DSMM-20 study (NCT05695508), which combines sequential targeting of BCMA and GPRC5D for unprecedented depth of response in patients with newly diagnosed MM [15].

## Supplementary information

Supplementary Material

## Data Availability

All data are displayed in respective sections of this manuscript and will be shared as source data by the corresponding author upon reasonable request.
