# Supplementary material for: Plasmapheresis facilitates soluble BCMA clearance and contributes to reversing primary resistance to anti-BCMA immunotherapy in multiple myeloma
Source: Leukemia. 2025 Sep 10;39(10):2563–7. doi: 10.1038/s41375-025-02757-6 (PMC12463651; doi:10.1038/s41375-025-02757-6)
Supplement: Supplementary file 1 — Supplementary Material [file 41375_2025_2757_MOESM1_ESM.pdf]

Plasmapheresis facilitates soluble BCMA clearance and contributes to reversing primary resistance to anti-BCMA immunotherapy in multiple myeloma *(R1 version)*

Authors:
Sven Neubert[1*], Umair Munawar[1*], Julia Mersi[1], Julia Noderer[1], Silvia Nerreter[1], Shilpa Kurian[1], Seungbin Han[1], Christina Verbruggen[1], Emma Besant[1], Nina Rein[1], Max Köppel[1], Johanna Lehmann[1], Tabea Köhler[1], Hannah Labinsky[1], Sigrun Häusl[1], Yoko Tamamushi[1], Xiang Zhou[1], Jule Pinter[2], Anna Laura Herzog[2], Kai Lopau[2], Elion Hoxha[2], Christoph Rummelt[3], Elena Gerhard-Hartmann[4], Andreas Rosenwald[4], Torsten Steinbrunn[1], Thomas Nerreter[1], Michael Hudecek[1], Hermann Einsele[1], Leo Rasche[1,5], K. Martin Kortüm[1,†], Johannes M. Waldschmidt[1,6,†]

SUPPLEMENT

Supplementary Figure 1

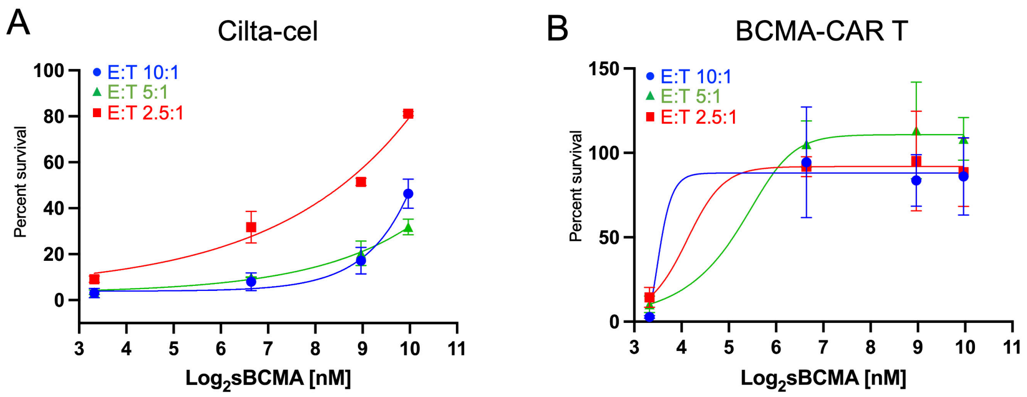

Suppl. Figure 1. sBCMA impairs the cytolytic activity of anti-BCMA CAR T-cells. OPM-2 cells were co-cultured with (A) cilta-cel or (B) BCMA-CAR T-cells at different E:T cell ratios. Soluble BCMA was titrated across different conditions (0-1000 nM) and cytolytic activity was measured using a luminescence-based viability assay. Data for each condition were normalized to Mock CAR T-cells at the corresponding E:T ratio. *__Abbreviations__: cilta-cel= ciltacabtagene autoleucel, BCMA= B-cell maturation antigen, CAR=chimeric antigen receptor, E:T= effector:target*
